# Discovery of Lignans from the Herbs of *Peperomia heyneana* with Inhibitory Activities on BPH-1 Cells

**DOI:** 10.3390/molecules30081809

**Published:** 2025-04-17

**Authors:** Yufei Xi, Juan Liu, Congcong Gao, Mingxuan Zhu, Baomin Feng, Xuan Lu

**Affiliations:** 1 College of Life and Health, Dalian University, Dalian 116622, China; xyf_1126@163.com (Y.X.); liujuan@163.com (J.L.); gcc1125@163.com (C.G.); zmx_214@163.com (M.Z.); fbmdlu@163.com (B.F.); 2 Dalian Marine Traditional Chinese Medicine Research Institute, Dalian 116622, China; 3 State Key Laboratory for Quality Ensurance and Sustainable Use of Dao-di Herbs, Beijing 100700, China

**Keywords:** *Peperomia heyneana*, lignans, BPH, structure elucidation

## Abstract

Chemical investigation on the whole herb of *Peperomia heyneana* Miq. resulted in the isolation of six lignans, including two previously undescribed compounds, named peperomianan A and B (**1**–**2**), and four known compounds, 1,2-cyclobutanedicarboxylic acid (**3**), (+)-medioresinol (**4**), (+)-pinoresinol (**5**), and (+)-yangambin (**6**). Their structures were established by extensive spectroscopic analyses. The absolute configuration of compound **1** was determined by comparison of the experimental and calculated electronic circular dichroism (ECD) spectra. Subsequently, the effects of all isolates on BPH-1 cells were evaluated in vitro by MTT assay.

## 1. Introduction

Benign prostate hyperplasia (BPH) is a common urinary disorder in men, typically presenting with symptoms such as prostate enlargement, bladder outlet obstruction, lower urinary tract symptoms, and dysuria [1]. There are many drugs that can be used to treat BPH [2], such as 5α-reductase inhibitors and α-blockers; however, these drugs have many side effects, such as headaches and weakness [3], so it is crucial to look for drugs with lower side effects from natural plants.

*Peperomia ruiz & pav*. is one of the largest genera of angiosperms, with approximately 1600 species [4], usually consisting of perennial herbaceous plants with a pantropical distribution of species, with maximum biodiversity in the neotropics [5]. Previous studies on peperomia genus resulted in the isolation of different types of chemical constituents, such as lignans [6,7], polyketides [8], benzopyranones [9], benzopyrans [10], benzoquinones [11], and phenylpropanoids [12], which have a variety of biological activities, including antimicrobial [13], anti-inflammatory [14], and antitumor [15,16].

Lignans are widely found in plants of the *Peperomia ruiz & pav*. and have a variety of biological activities, such as anti-prostate cancer [17], antitumor [18], anti-inflammatory [19], anti-trypanosomia [20], and other activities. Lignans have previously been identified from the *Peperomia heyneana* Miq. of this genus and anti-HIV activity of the compounds has been evaluated [21], but no studies have been done related to BPH disease. Therefore, we conducted phytochemical studies and studied the activity of the plant against BPH using the MTT (3-(4,5-dimethylthiazol-2-yl)-2,5-diphenyltetrazolium bromide) assay, as a result, six lignans were obtained, several of which showed good biological activity against BPH diseases.

## 2. Results and Discussion

Peperomianan A (**1**), brown oil, [α]_20__D_ −10.0 (c 0.1, MeOH), has a molecular formula of C_19_H_24_O_6_ (8 degrees of unsaturation) based on its HRESIMS (high-resolution electrospray ionization mass spectroscopy) data ([M + Na]^+^ at *m*/*z* 371.1466, calcd for C_19_H_24_O_6_Na, 371.1471). The ^1^H NMR spectrum and HSQC (heteronuclear single quantum correlation) NMR spectroscopic data (Table 1) suggested the presence of a 1,2,4,5-tetrasubstituted benzene ring at *δ*_H_ 6.63 (1H, s, H-3), 6.94 (1H, s, H-6), a 1,2,4,6-tetrasubstituted benzene ring at *δ*_H_ 6.09 (1H, br d, *J* = 2.5 Hz, H-3′), 6.04 (1H, br d, *J* = 2.5 Hz, H-5′), three hydroxyl protons at *δ*_H_ 10.25 (1H, s, 2′-OH), 8.90 (1H, br s, 4′-OH), 5.86 (1H, br s, 8-OH), three methoxy groups at *δ*_H_ 3.54 (3H, s, 5-OCH_3_), 3.75 (3H, s, 4-OCH_3_), 3.78 (3H, s, 2-OCH_3_), two methyl groups at *δ*_H_ 1.03 (3H, d, *J* = 6.0 Hz, H-9), 2.06 (3H, s, H-7′) as well as two methines at *δ*_H_ 4.39 (1H, d, *J* = 5.0 Hz, H-7), 4.43 (1H, m, H-8). The ^13^C NMR of **1** (Table 1) displayed 19 carbon signals corresponding to twelve aromatic carbons (*δ*_C_ 156.8, 155.9, 150.8, 147.8, 142.1, 139.2, 122.2, 116.5, 115.8, 109.0, 101.9, 98.1), two methine carbons (*δ*_C_ 67.5 and 44.4), two methyl carbons (*δ*_C_ 22.0 and 21.0) and three methoxy carbons (*δ*_C_ 56.5, 56.3 and 55.8).

**Figure 1:**
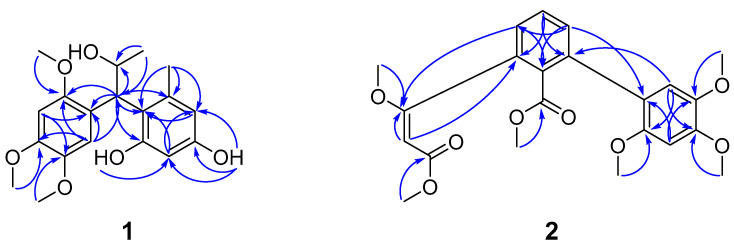
Key HMBCs for compounds **1** and **2**.

The HMBCs (heteronuclear multiple bond correlations) (Figure 1) of H-7/C-1, C-2, C-8, C-1′, C-2′ and C-6′, H-9/C-7 and C-8 indicated a 1,2,4,5-tetrasubstituted benzene ring shared a common atom at C-7 (*δ*_C_ 44.4) with the 1,2,4,6-tetrasubstituted benzene ring. The presence of propan-2-ol moiety was supported by the HMBCs of H-9/C-7 and C-8. The methyl group was located at C-6′ on the basis of the correlations of H-7′/C-1′, C-5′, and C-6′. The correlations from the methoxy protons (*δ*_H_ 3.78) to C-2, the methoxy protons (*δ*_H_ 3.75) to C-4 along with the methoxy protons (*δ*_H_ 3.54) to C-5 demonstrated a 2,4,5-trimethoxyphenyl moiety. The 6-methylbenzene-2,4-diol moiety was unambiguously confirmed by the cross-peaks of hydroxyl protons (*δ*_H_ 10.25)/C-3′, *δ*_H_ 8.90/C-3′, C-4′ and C-5′. The relative configurations of **1** can be demonstrated by the small coupling constant of *J*_H-7, H-8_ = 5.0 Hz.

**Figure 2:**
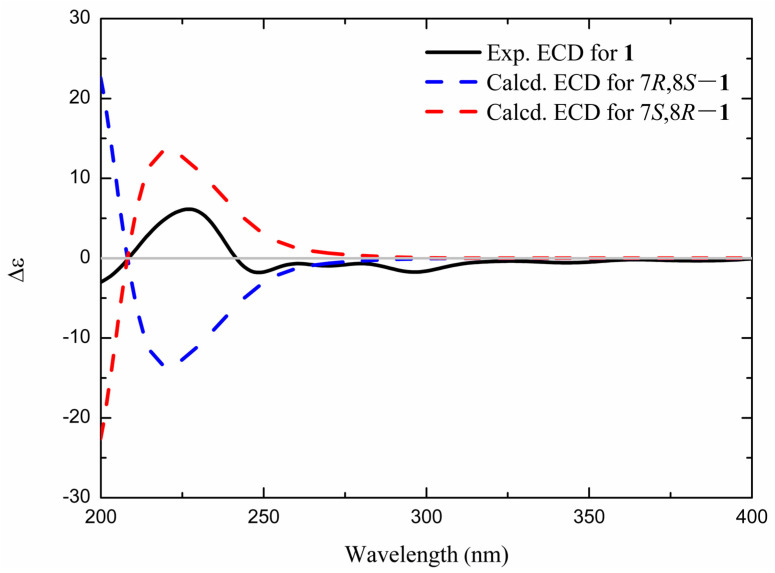
Calculated and experimental ECD spectra of **1**.

The absolute configuration of **1** was determined by comparing the experimental ECD (electronic circular dichroism) spectrum with those predicted from quantum mechanical TDDFT (time-dependent density functional theory) calculations. As shown in Figure 2, the calculated ECD of *7S*, *8R*-**1** matched with the experimental ECD of **1**. Thus, **1** was assigned as peperomianan A.

Peperomianan B (**2**) was isolated as a yellow-green solid and its molecular formula of C_22_H_24_O_8_ was analyzed by the HR-ESI-MS at *m*/*z* 439.1361 [M + Na]^+^ (Calcd. for C_22_H_24_O_8_Na, 439.1369), accounting for eleven double bond equivalents (DBEs). The ^1^H NMR spectrum and HSQC NMR spectroscopic data (Table 1) suggested the presence of a 1,2,3-trisubstituted benzene ring at *δ*_H_ 7.39 (1H, d, *J* = 7.7 Hz, H-4), 7.48 (1H, t, *J* = 7.7 Hz, H-5), 7.29 (1H, d, *J* = 7.7 Hz, H-6), a 1,2,4,5-tetrasubstituted benzene ring at *δ*_H_ 6.70 (1H, s, H-3′), 6.73 (1H, s, H-6′), an olefinic proton at *δ*_H_ 5.36 (1H, s, H-2″) together with six methoxy groups at *δ*_H_ 3.71 (3H, s, 1″-OCH_3_), 3.48 (3H, s, 3″-OCH_3_), 3.51 (3H, s, 7-OCH_3_), 3.83 (3H, s, 2′-OCH_3_), 3.63 (3H, s, 4′-OCH_3_), and 3.71 (3H, s, 5′-OCH_3_).

Analysis of the ^13^C NMR data (Table 1) revealed 22 carbon signals including a 1,2,3-trisubstituted aromatic moiety (*δ*_C_ 137.0, 134.2, 132.2, 132.1, 129.0, 128.5), a 1,2,4,5-tetrasubstituted aromatic moiety (*δ*_C_ 150.0, 149.2, 142.5, 119.6, 114.7, 97.8), two carbonyl carbons (*δ*_C_ 167.6, 165.9), two olefinic carbons (*δ*_C_ 170.6, 92.5) as well as six methoxy carbons (*δ*_C_ 56.9, 56.2, 55.8, 55.7, 51.5, 50.5).

**Figure 3:**
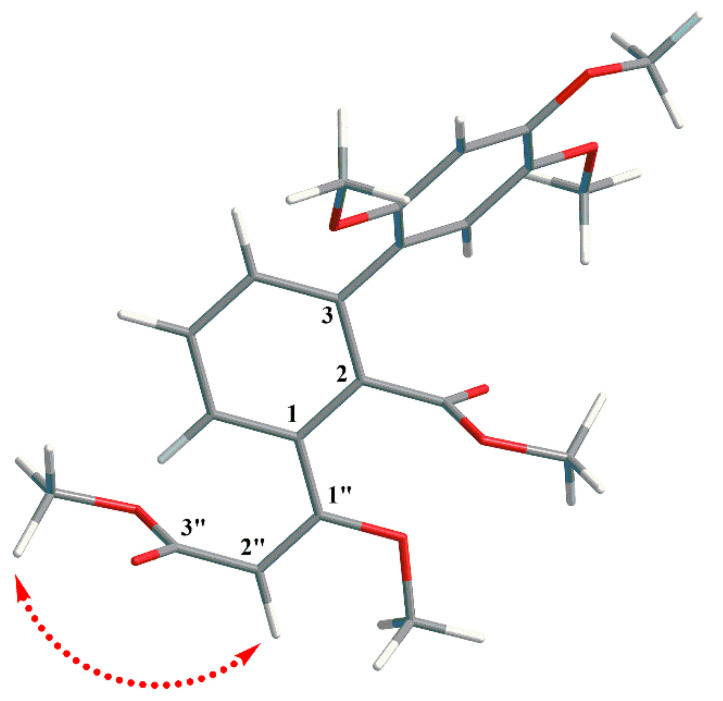
Key NOESY correlation (arrows, from ^1^H to ^1^H) of compound **2**.

The 1,2,3-trisubstituted benzene ring was bound to a methyl-3-methoxybut-2-enoate moiety at C-1″, which was supported by the HMBCs (Figure 1) of H-6/C-1″, H-2″/C-1″ and C-1, the methoxy protons (*δ*_H_ 3.71)/C-1″, and the methoxy protons (*δ*_H_ 3.48)/C-3″. The correlations of H-4/C-1′ and H-6′/C-3 determined the C-3 of the 1,2,3-trisubstituted benzene ring was linked to the C-1′ of the 1,2,4,5-tetrasubstituted benzene ring. In addition, the correlations of methoxy protons (*δ*_H_ 3.51)/C-7 and the methoxy protons (*δ*_H_ 3.71)/C-5′, *δ*_H_ 3.63/C-4′, *δ*_H_ 3.83/C-2′ illustrated the location of each methoxy group. The NOESY (nuclear overhauser effect spectroscopy) correlations (Figure 3) were carried out to determine the configuration of a double bond between C-1″ and C-2″, which was confirmed to be *E*-configuration based on the correlation of H-2″ with *δ*_H_ 3.71 (1″-OCH_3_). Consequently, the aforementioned analysis allowed the determination of the gross structure of **2**, which was given the trivial name peperomianan B.

**Figure 4:**
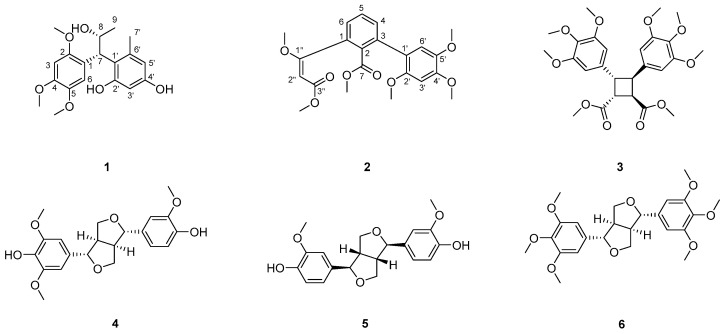
The structures of compounds **1**–**6**.

In addition, four known lignans (**3**–**6**) were isolated from *P. heyneana* and defined as 1,2-cyclobutanedicarboxylic acid (**3**) [22], (+)-medioresinol (**4**) [23], (+)-pinoresinol (**5**) [24], and (+)-yangambin (**6**) [25], respectively (Figure 4).

**Figure 5:**
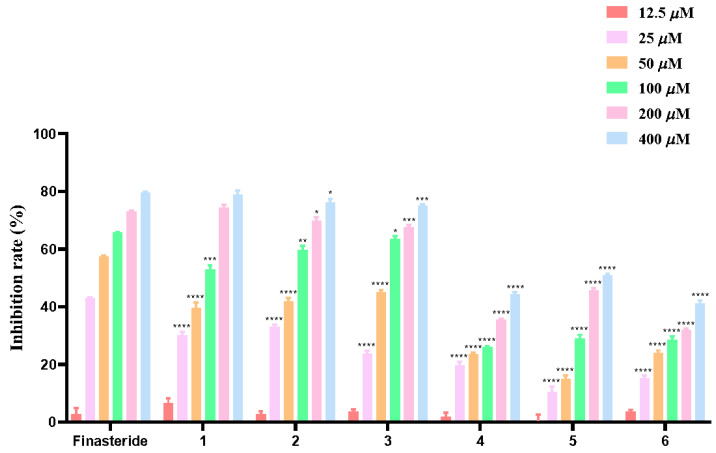
Cytotoxicity assay of compounds **1–6** on BPH-1 cells. Cell viabilities were determined by MTT assay in the presence of positive control (Finasteride) and the tested compounds at different concentrations (12.5, 25, 50, 100, 200, 400 μM). * *p* < 0.05, ** *p* < 0.01, *** *p* < 0.001, **** *p* < 0.0001 compared with positive control.

All the isolates were evaluated for their effects on BPH-1 cells by MTT assay. A positive drug, finasteride, which is very effective against BPH disease, was used as a positive control. As shown in Figure 5, the inhibitory effects of tested compounds on BPH-1 cell proliferation were dose-dependent within a certain concentration range.

The IC_50_ values of the test compounds were calculated. The results showed that the IC_50_ values of compounds **1**, **2**, and **3** were close to that of the positive drug-finasteride (IC_50_ = 62.96 μM), being 82.10, 82.40, and 85.36 μM, respectively (Table 2), indicating that among the test compounds, they exhibit better effects on BPH-1 cells. Among the tested compounds, **1** showed better inhibitory effects in vitro at the concentration of 12.5 μM, while the positive control finasteride showed an inhibition rate of 2.63% at the same concentration.

## 3. Materials and Methods

### 3.1. General Experimental Procedures

The Fresh whole herb of *Peperomia heyneana* Miq. was freeze-dried in a freeze dryer (Lyovapor L-200, BUCHI, Flawil, Switzerland). The organic solvents were distilled prior to the separation process. The NMR spectra were recorded by Bruker Avance II 500M nuclear magnetic resonance spectrometers (Bruker AvanceII, Woltersbach, Berlin, Germany). HRESIMS were acquired on an AB Sciex QTOF 4600 mass spectrometer (AB Sciex, Boston, MA, USA) in positive-ion mode. Optical rotation values were measured on an Autopol IV digital polarimeter (Autopol IV, Rudolph Research Analytica, Hackettstown, NJ, USA). ECD spectra were conducted on a Bio-Logic MOS 450 detector (Bio-Logic, Grenoble, France). HPLC was performed on a system composed of Agilent 1260 (XB C18 10 mm × 250 mm, 5 μM) (Agilent Technologies Inc., Santa Clara, CA, USA) and Shimadzu LC-20AR (XB C18 10 mm × 250 mm, 5 μm) (Shimadzu, Kyoto, Japan). Gel LH-20 (Sephadex, Sweden) and silica gel (Qingdao Ocean Chemical Co., Ltd., Qingdao, China) were used for column chromatography (CC). TLC was performed on precoated silica gel GF254 (Qingdao Marine Chemistry Co., Ltd., Qingdao, China) plates. MTT assays were performed on a Varioskan Flash Multimode Reader (Thermo Scientific Co., Waltham, MA, USA). BPH-1 cell lines were obtained from Applied Biological Materials Inc. (Zhenjiang, China). Fetal bovine serum (FBS) was purchased from Gibco Company (Grand Island, NY, USA).

### 3.2. Plant Material

The whole herb of *Peperomia heyneana* Miq. was collected from Mengzi City (Yunnan Province) in China in November 2022 and authenticated by Professor Sheng-Ji Pei (Kunming Institute of Botany, Chinese Academy of Sciences, China). A voucher specimen (No. 20221128) of the plant is stored at the School of Life and Health, Dalian University.

### 3.3. Extraction and Isolation

The fresh whole plants of *Peperomia heyneana* Miq. (4.0 kg) were dried using a freeze dryer and ground to powder form (456.4 g). The powdered material was extracted with 95% (*v*/*v*) industrial EtOH (ethyl alcohol) three times (3 h/time), and the ethanol liquid (1 kg) was collected. After filtering out the residue, the extract was combined and concentrated under reduced pressure to get the EtOH extract (96 g). The EtOH extract was suspended in hot water and extracted with PE (petroleum ether), EtOAc (ethyl acetate), and *n*-BuOH (*n*-Butanol) six times to obtain PE extract (15.4 g), EtOAc extract (11.4 g), and *n*-BuOH extract (34.4 g), respectively. The EtOAc extract was separated by silica gel CC (CH_2_Cl_2_/MeOH, 100:0–0:100, *v*/*v*) to obtain fractions 1–9, according to the results of TLC, and combined with the same fractions. Fraction 3 was prepared by semi-preparative HPLC (MeOH/H_2_O, 35:65, flow rate 3.0 mL/min, detection wavelength UV at 210 nm) to yield compound **1** (1.2 mg, *t*_R_ = 20 min). Fractions 1 and 2 were prepared by semi-preparative HPLC (MeOH/H_2_O, 45:55, flow rate 3.0 mL/min, detection wavelength UV at 210 nm) to afford compounds **4** (5.4 mg, *t*_R_ = 30 min) and **5** (6.0 mg, *t*_R_ = 28 min). Fraction 5 was prepared by semi-preparative HPLC (CH_3_CN/H_2_O, 48:52, flow rate 3.0 mL/min, detection wavelength UV at 210 nm) to yield compounds **2** (1.4 mg, *t*_R_ = 25 min) and **6** (1.5 mg, *t*_R_ = 35 min). Fraction 6 was separated by semi-preparative HPLC (CH_3_CN/H_2_O, 45:55, flow rate 3.0 mL/min, detection wavelength UV at 210 nm) to acquire compound **3** (1.2 mg, *t*_R_ = 15 min).

Peperomianan A (**1**): Brown oil, [α]_20__D_ −10.0 (c 0.1, MeOH); UV (MeOH) *λ*_max_ (log *ε*) 209.0 (2.59) nm; ECD (MeOH) *λ*_max_ (Δ*ε*) 194 (−3.59), 227 (+6.14) nm. ^1^H and ^13^C NMR data see Table 1; HRESIMS: *m*/*z* 371.1466 [M + Na]^+^ (calcd for C_19_H_24_O_6_Na, 371.1471).

Peperomianan B (**2**): Yellow-green solid. [α]_20__D_ −2.0 (c 0.1, MeOH); UV (MeOH) *λ*_max_ (log *ε*) 215.0 (3.53) nm; ^1^H and ^13^C NMR data see Table 1; HRESIMS: *m*/*z* 439.1361 [M + Na]^+^ (calcd for C_22_H_24_O_8_Na, 439.1369).

### 3.4. ECD Calculations

Conformational analysis of compound **1** was carried out with the MMFF94 force field in CONFLEX software (version: CONFLEX 9). All the conformers obtained were screened based on the energy of optimized structures at the B3LYP/6-31G(d) level with an energy window of 10 kcal/mol on the Gaussian 09 program package [26]. Then, the theoretical ECD calculations of the conformations of compound **1** were performed by the TDDFT method at the B3LYP/6-311++G (2d, *p*) level with the CPCM (conductor-like polarizable continuum model) model in methanol solution. Finally, the calculated ECD curve was generated by SpecDis 1.51 [27].

### 3.5. Cell Culture

BPH-1 cell lines were obtained from Applied Biological Materials Inc. (Zhenjiang, China) and cultured in RPMI-1640 medium (Hyclone, Logan, UT, USA), which was supplemented with 10% fetal bovine serum (FBS, Gibco, Gaithersburg, MD, USA) and 1% bispecific antibodies (Gibco, Gaithersburg, UD, USA) in a humidified atmosphere containing 5% CO_2_ at 37 °C. Logarithmically growing cells were used in all the experiments.

### 3.6. Cytotoxicity Assay and Statistical Analysis

The effect of all isolated compounds against BPH-1 cells was examined following the reported procedures [28]. In brief, antiproliferative of the compounds were investigated by MTT(3-(4,5-dimethylthiazol-2-yl)-2,5-diphenyltetrazolium bromide)-based cell viability assays. Obtain well-grown BPH-1 cells in the logarithmic phase from the cell culture incubator, discard the old culture medium in the culture flask and wash with PBS (2 mL), discard, add trypsin (1.5 mL) to digest for 2 min, add RPMI-1640 complete medium (3 mL, 10% fetal bovine serum, 1% bispecific antibody) and centrifuge to separate, seed the cells into a 96-well plate (NEST, Wuxi, China), control the number of cells at 5 × 10^3^ per well, 100 μL culture medium per well, After plating the cells, they are placed in the incubator and cultured for 24 h until the cells are fully adherent. Prepare a certain amount of compounds **1**, **2,** and positive drug (Finasteride), based on 20 μL DMSO stock solutions, the compounds were serially diluted in standard growth media to reach final concentrations of 400 μM, 200 μM, 100 μM, 50 μM, 25 μM, and 12.5 μM for cell treatment, data were determined in setting up 6 concentration gradients and 6 replicates, continue to culture for 24 h after sequential dosing.

For the MTT assay, add 20 μL of MTT solution (5 mg/mL) to each well in the dark, and continue to incubate for 4 h, after discarding the MTT solution, DMSO was added in order to dissolve the formed formazan, followed by measuring formazan absorbance with a microplate reader (Varioskan LUX, Thermo Fisher Scientific, Bend, OR, USA) at 490 nm, then calculate the growth inhibition rate and IC_50_ of the cells according to the obtained absorbance (OD). The results are shown as a percentage of the control values obtained from untreated cultures, i.e., cell viability in percent.

All results and data were confirmed in at least three separate experiments. Data are expressed as means ± SD. The level of statistical significance was determined by analysis of one-way ANOVA using GraphPad Prism 6 from GraphPad Software (San Diego, CA, USA). *p* < 0.05 was considered statistically significant.

## 4. Conclusions

In conclusion, six lignans including two undescribed ones were isolated from aerial parts of *P. heyneana*. Their structures were determined by extensive NMR and HRESIMS analyses. The absolute configuration of **1** was defined with the help of the comparison of the experimental and calculated ECD spectra. Furthermore, the anti-BPH effects of all isolates were evaluated in vitro by MTT assay. As a result, the inhibitory effect of all tested compounds increases in a dose-dependent manner. In addition, the inhibition rates of compounds **1**, **3,** and **6** were higher than that of the positive control at the concentration of 12.5 μM. The IC_50_ values of compounds **1**, **2**, and **3** were 82.10, 82.40, and 85.36 μM, respectively, which were relatively close to that of the positive drug finasteride (IC_50_ = 62.96 μM), compared with the other three compounds, they showed better inhibitory effects on BPH-1 cells. The study provided new insight into the development and utilization of *P. heyneana* in anti-BPH medicinal products.

## Figures and Tables

**Table 1:** ^1^H (500 MHz) and ^13^C NMR (125 MHz) spectroscopic data of **1** and **2** in DMSO-*d*_6_(Dimethyl sulfoxide-d6).

Position	1		2	
	*δ*_H_ (Multi, *J* in Hz)	*δ*_C_	*δ*_H_ (Multi, *J* in Hz)	*δ*_C_
1	‒	122.2	‒	134.2
2	‒	150.8	‒	132.1
3	6.63, s	98.1	‒	137.0
4	‒	147.8	7.39, d (7.7)	132.2
5	‒	142.1	7.48, t (7.7)	129.0
6	6.94, s	115.8	7.29, d (7.7)	128.5
7	4.39, d (5.0)	44.4	‒	167.6
8	4.43, m	67.5	‒	‒
9	1.03, d (6.0)	22.0	‒	‒
1′	‒	116.5	‒	119.6
2′	‒	156.8	‒	149.2
3′	6.09, br d (2.5)	101.9	6.70, s	97.8
4′	‒	155.9	‒	150.0
5′	6.04, br d (2.5)	109.0	‒	142.5
6′	‒	139.2	6.73, s	114.7
7′	2.06, s	21.0	‒	‒
1″	‒	‒	‒	170.6
2″	‒	‒	5.36, s	92.5
3″	‒	‒	‒	165.9
2-OCH_3_	3.78, s	55.8	‒	‒
4-OCH_3_	3.75, s	56.3	‒	‒
5-OCH_3_	3.54, s	56.5	‒	‒
7-OCH_3_	‒	‒	3.51, s	51.5
2′-OCH_3_	‒	‒	3.83, s	55.8
4′-OCH_3_	‒	‒	3.63, s	55.7
5′-OCH_3_	‒	‒	3.71, s	56.2
1″-OCH_3_	‒	‒	3.71, s	56.9
3″-OCH_3_	‒	‒	3.48, s	50.5
8-OH	5.86, br s	‒	‒	‒
2′-OH	10.25, s	‒	‒	‒
4′-OH	8.90, br s	‒	‒	‒

**Table 2:** Cytotoxic activity of isolated compounds (**1**–**6)** from *P. heyneana* on benign prostatic hyperplasia cells (BPH-1).

Compounds	IC_50_ (μM)
Finasteride	62.96 ± 1.10
**1**	82.10 ± 2.21
**2**	82.40 ± 0.79
**3**	85.36 ± 1.63
**4**	218.00 ± 3.47
**5**	216.06 ± 1.69
**6**	229.49 ± 2.54

Note: Data are expressed as means ± SD.

## Data Availability

The diverse data generated and analyzed during this work are available from the corresponding author on request.

## Abbreviations

MTT	3-(4,5-dimethylthiazol-2-yl)-2,5-diphenyltetrazolium bromide
HRESIMS	High-Resolution Electrospray Ionization Mass Spectroscopy
HSQC	Heteronuclear Single Quantum Correlation
DMSO-*d*_6_	Dimethyl sulfoxide-*d*_6_
HMBC	Heteronuclear Multiple Bond Correlation
ECD	Electronic Circular Dichroism
TDDFT	Time-Dependent Density Functional Theory
NOESY	Nuclear Overhauser Effect Spectroscopy
PE	Petroleum Ether
EtOAc	Ethyl acetate
*n-*BuOH	*n*-Butanol
Fr.	Fractions
